# Apremilast exerts protective effects on stroke outcomes and blood–brain barrier (BBB) dysfunction through regulating Rho‐associated protein kinase 2 expression

**DOI:** 10.1002/brb3.2677

**Published:** 2022-08-15

**Authors:** Mingyuan Wang, Xiangyuan Meng, Zhihua Cheng

**Affiliations:** ^1^ Department of Neurology People's Hospital of Xinjiang Uygur Autonomous Region Urumqi 830002 China; ^2^ Xinjiang Clinical Research Center for Stroke and Neurological Rare Disease People's Hospital of Xinjiang Uygur Autonomous Region Urumqi Xinjiang Uygur Autonomous Region, 830002 China; ^3^ Department of Neurology People's Hospital of Yicheng District Zaozhuang China; ^4^ Department of Neurosurgery, Shanghai Ninth People's Hospital Shanghai Jiao Tong University School of Medicine Shanghai China

**Keywords:** apremilast (APR), blood–brain barrier (BBB), Claudin‐5, endothelial permeability, ROCK2, stroke

## Abstract

**Aims:**

Stroke is a devastating event and a huge public health concern worldwide. Apremilast (APR) is a selective inhibitor of phosphodiesterase‐4 involved in various neurological diseases, including stroke. However, the protective effects of APR on stroke have not been investigated. Here, we explored the effects of APR on stroke outcomes and blood–brain barrier (BBB) dysfunction using a middle cerebral artery occlusion (MCAO) stroke mice model.

**Results:**

The results show that APR attenuated neurological injury in MCAO mice with decreased neurological deficit scores and infarct size, as well as increased hanging grip time. The increased BBB permeability and decreased expression of the tight junction protein Claudin‐5 in MCAO mice were attenuated by APR treatment. APR treatment also mitigated neuroinflammation in MCAO mice, as shown by the decreased levels of inflammatory cytokines. In vitro assays also proved that APR ameliorated the oxygen/glucose deprivation/reoxygenation (OGD/R)‐induced increase in endothelial permeability and restored the expression of Claudin‐5 in bEnd.3 brain endothelial cells. Moreover, overexpression of ROCK2 in bEnd.3 cells abolished the protective effects of APR on endothelial permeability against OGD/R induction.

**Conclusion:**

Taken together, our results demonstrate that APR showed significant efficacy on ischemic stroke outcomes by alleviating enhanced BBB permeability and neuroinflammation by inhibiting ROCK2. These findings suggest a novel therapeutic window for ischemic stroke.

## Graphical abstract

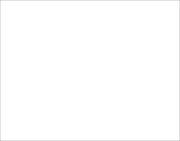

## 1 INTRODUCTION

Stroke is a devastating event that remains the second leading cause of death worldwide, continues to have a growing prevalence, and is a huge public health concern (Feigin et al., 2017). More than 85% of stroke cases are ischemic strokes, which are characterized by impaired blood supply. The reduced delivery of oxygen, glucose, and other essential nutrients results in irreversible brain injury (Maida et al., 2020). Associated mechanisms involved in ischemic stroke are critical for developing novel therapeutics.

Blood–brain barrier (BBB) is a unique structure that controls cerebral homeostasis by limiting and regulating the exchange of exogenous, endogenous xenobiotics and associated metabolites between the blood and the brain (Abbott et al., 2010). BBB dysfunction plays a central role in the development of brain injury and subsequent neurological dysfunction in ischemic stroke (Jiang et al., 2018). Following a stroke, BBB damage is mainly characterized by loss of tight junction integrity with altered expression of associated proteins. These modulations cause increased paracellular permeability, which finally leads to severe pathological consequences, such as vasogenic edema formation, hemorrhagic transformation, and mortality (Sandoval & Witt, 2008). Therefore, protecting against BBB dysfunction is critical in the setting of ischemic stroke patients.

Phosphodiesterase‐4 (PDE4) is an intracellular nonreceptor enzyme that is mainly present in epithelial cells, immune cells, and brain cells (Bhat et al., 2020). It has been identified to modulate inflammation and epithelial integrity by regulating cyclic adenosine monophosphate (cAMP) and subsequent genes and proteins (Lynch et al., 2006). Thus, targeting PDE4 is a promising approach to controlling dermatological, pulmonary, and neurological diseases. Numerous PDE4 inhibitors have been approved for the treatment of inflammatory psoriatic arthritis, airway diseases, and atopic dermatitis (Li et al., 2018). Over the past decade, several PDE4 inhibitors have been found to have protective effects against ischemic stroke (Wang et al., 2018). Apremilast (APR) is a selective PDE4 inhibitor used for the efficacious treatment of moderate‐to‐severe plaque psoriasis, which has been demonstrated to be a chronic inflammatory skin disease (Chimenti et al., 2015). Otto M et al. reported that APR inhibited the expression of interleukin 6 (IL‐6) and monocyte chemoattractant protein‐1 (MCP‐1) in IL‐17A‐challenged human umbilical vein endothelial cells. Importantly, they found that APR suppressed the activation of nuclear factor‐κB and mitogen‐activated protein kinases signaling pathways (Otto et al., 2021). However, the protective effects of APR on stroke have not been reported. Here, we aimed to explore the effects of APR on stroke outcomes, BBB dysfunction, and associated mechanisms using both in vivo and in vitro assays.

## 2 MATERIALS AND METHODS

### 2.1 Animal experiments and treatment

A total of 60 male C57BL/6 mice (8 weeks old, Charles River Laboratories, Beijing, China) were divided into four groups (15 mice per group): the sham group; APR treatment group, 5 mg·kg^−1^·day^−1^ of APR was injected at 0, 6, and 18 h after sham surgery; middle cerebral artery occlusion (MCAO) model group, subjected to MCAO surgery; MCAO+ APR treatment group, 5 mg·kg^−1^·day^−1^ of APR was injected at 0, 6, and 18 h post‐MCAO surgery.

The transient MCAO models were established as described previously (Pu et al., 2016). Briefly, a skin incision was made along the midline of the mouse neck to expose the internal common carotid artery (ICA) and external common carotid artery. MCAO was induced for 1 h in ICA using an intraluminal filament (size 5–0, Doccol Corporation, Sharon, MA, USA). After occlusion, mice were subjected to reperfusion by filament withdrawal. Mice in the sham group underwent a sham‐operated MCAO surgery, and the filament was immediately removed after insertion.

### 2.2 Measurement of neurological deficit

Neurological status was assessed 48 h post‐MCAO to estimate the degree of brain injury according to an acceptable five‐point scoring method (Broughton et al., 2014).

### 2.3 Hanging grip test

Mice were placed on grip wire (25 cm length, 1.0 mm diameter) above a soft ground with their four paws (Ji et al., 2009). The amount of hanging time that mice clung to the wire was recorded. The test was then ended for mice that could hang for a maximum duration time of 600 s.

### 2.4 Determination of infarct size

The brain tissues were removed, cut into 2 mm coronal sections, and stained with 2% TTC (Sigma, St. Louis, MO, USA) at 37°C for 30 min. Then, the tissues were fixed in 10% formalin, and images were captured. The digitized images of each brain section were analyzed using computerized ImageJ analysis software in a double‐blinded manner.

### 2.5 Evaluation of BBB permeability

Evans blue and sodium fluorescein were employed to evaluate BBB permeability (Yen et al., 2013). Seven days after MCAO, mice were anesthetized using 2% pentobarbital sodium, followed by injection with Evans blue solution or sodium fluorescein (20 mg/kg of body weight) via the tail vein. After 24 h, the ipsilateral hemisphere was collected for the preparation of homogenate using trichloroacetic acid. After centrifugation, the supernatants were collected for the photospectrometric determination of Evans blue and sodium fluorescein fluorescence.

### 2.6 Immunostaining

The cortex samples were collected and cut into slides for immunostaining (Kim et al., 2020). Briefly, the sections were prefixed in acetone, followed by rinsing with water. Each section was then exposed to a blocking solution for 1 h at 37°C, incubated with a primary antibody against Claudin‐5 (Abcam, Cambridge, MA; 1:500) overnight at 4°C, and incubated with a secondary antibody (1:500) for 2 h at 37°C. A microscope (Carl Zeiss, Oberkochen, Germany) was used to examine the sections.

### 2.7 Enzyme‐linked immunosorbent assay

Cortices homogenates obtained from experimental mice were prepared with cold phosphate‐buffered saline. IL‐6 and MCP‐1 levels in homogenates were estimated using enzyme‐linked immunosorbent assay (ELISA) kits: IL‐6 (#D6050; R&D Systems, USA) and MCP‐1 (#DCP00; R&D Systems).

### 2.8 Cell culture and treatment

bEnd.3 brain endothelial cells (Bioleaf Biotech Co., Ltd., Shanghai, China) were cultured in RPMI 1640 (Invitrogen, Carlsbad, CA, USA) supplemented with 15% fetal bovine serum (Sigma–Aldrich, USA), 100 U/ml penicillin, and 100 U/ml streptomycin (Sigma–Aldrich). For OGD/R administration, cells were incubated for 6 h with glucose‐free medium and maintained in a hypoxia chamber with an atmosphere of 1% O_2_, 5% CO_2_, and 94% N_2_, after which the cells were cultured under normoxia condition with an atmosphere of 5% CO_2_ and 95% air. For the OGD/R + APR treatment group, cells were pretreated with 5 μM APR and then subjected to OGD/R treatment.

After reaching a confluence of ∼70%–80%, bEnd.3 cells were infected with ROCK2 expression lentiviral vector (LV‐ROCK2; GeneChem Co., Ltd., Shanghai, China). Cells infected with the empty lentiviral expression vector (LV‐NC, GeneChem) were deemed the control group.

### 2.9 qRT‐PCR

The total RNAs were extracted from cortices and bEnd.3 cells using TRIzol reagent (Invitrogen). Then, the RNAs were used to synthesize cDNA with SuperScript III Reverse Transcriptase (Invitrogen). Maxima SYBR Green/ROX qPCR Master Mix (Thermo Fisher Scientific, MA, USA) was used for the real‐time quantitative (qRT‐PCR) on a PikoReal 96 Real‐Time PCR System (Thermo Fisher Scientific). The gene expression levels of IL‐6, MCP‐1, Claudin‐5, and ROCK2 were normalized to GAPDH. The following primers were used: IL‐6, forward 5′‐GAGGATACCACTCCCAACAGACC‐3′, reverse 5′‐AAGTGCATCATCGTTGTTCATACA‐3′; MCP‐1, forward 5′‐TTTTTGTCACCAAGCTCAAGAG−3′, reverse 5′‐CTGTGTAGCCATCTGTTGAGTT‐3′; ROCK2, forward 5′‐GGTTTACAGATGAAAGCGGAAGA−3′, reverse 5′‐GTGATGCCTTATGACGAACCAA‐3′; Claudin‐5, forward 5′‐TGGCACTCTTTGTTACCTTGACC‐3′, reverse 5′‐ACCGTTGGATCATAGAACTCCC‐3′; GADPH, forward 5′‐GAGTCAACGGATTTGGTCGT‐3′, reverse 5′‐GACAAGCTTCCCGTTCTCAG‐3′.

### 2.10 Western blot analysis

bEnd.3 cells were lysed with RIPA lysis buffer (Thermo Fisher Scientific), and then the samples were subjected to SDS‐PAGE gel separation and western blot analysis with antibodies against Claudin‐5 (#ab131259, Abcam, Cambridge, MA, USA,1:1000), ROCK2 (#ab125025, Abcam, 1:1000), β‐actin (#ab8226 Abcam, 1:1000), and a secondary antibody (#ab150077 Abcam, 1:3000) as previously described (Comajoan et al., 2018). Enhanced chemiluminescence detection reagent was also used to visualize the blots on the membranes.

### 2.11 Endothelial permeability

The permeability of bEnd.3 brain endothelial cells was assessed by detecting sodium fluorescein leakage using a chamber transwell insert as previously described (Cao et al., 2016). The concentration of sodium fluorescein in the lower chamber solution was determined using a fluorescence reader (Thermo Fisher, Waltham, MA, USA) with a reference wavelength of 485 nm (excitation) and 530 nm (emission).

### 2.12 Transendothelial electrical resistance

Measurement of transendothelial electrical resistance (TEER) on bEnd.3 brain endothelial cells after OGD/R treatment was performed using the ECIS system (Applied Biophysics, Troy, NY, USA) as previously described (Diaz‐Canestro et al., 2018). The final unit area resistance values are shown as Ω × cm^2^.

### 2.13 Statistical analysis

Statistical analysis was performed using GraphPad Prism 8 with analysis of variance followed by Dunnett's test. The data are presented as the mean ± standard deviation. *p*‐Values <.05 were considered statistically significant.

## 3 RESULTS

### 3.1 Effects of APR on brain injury in the MCAO mice model

**FIGURE 1:**
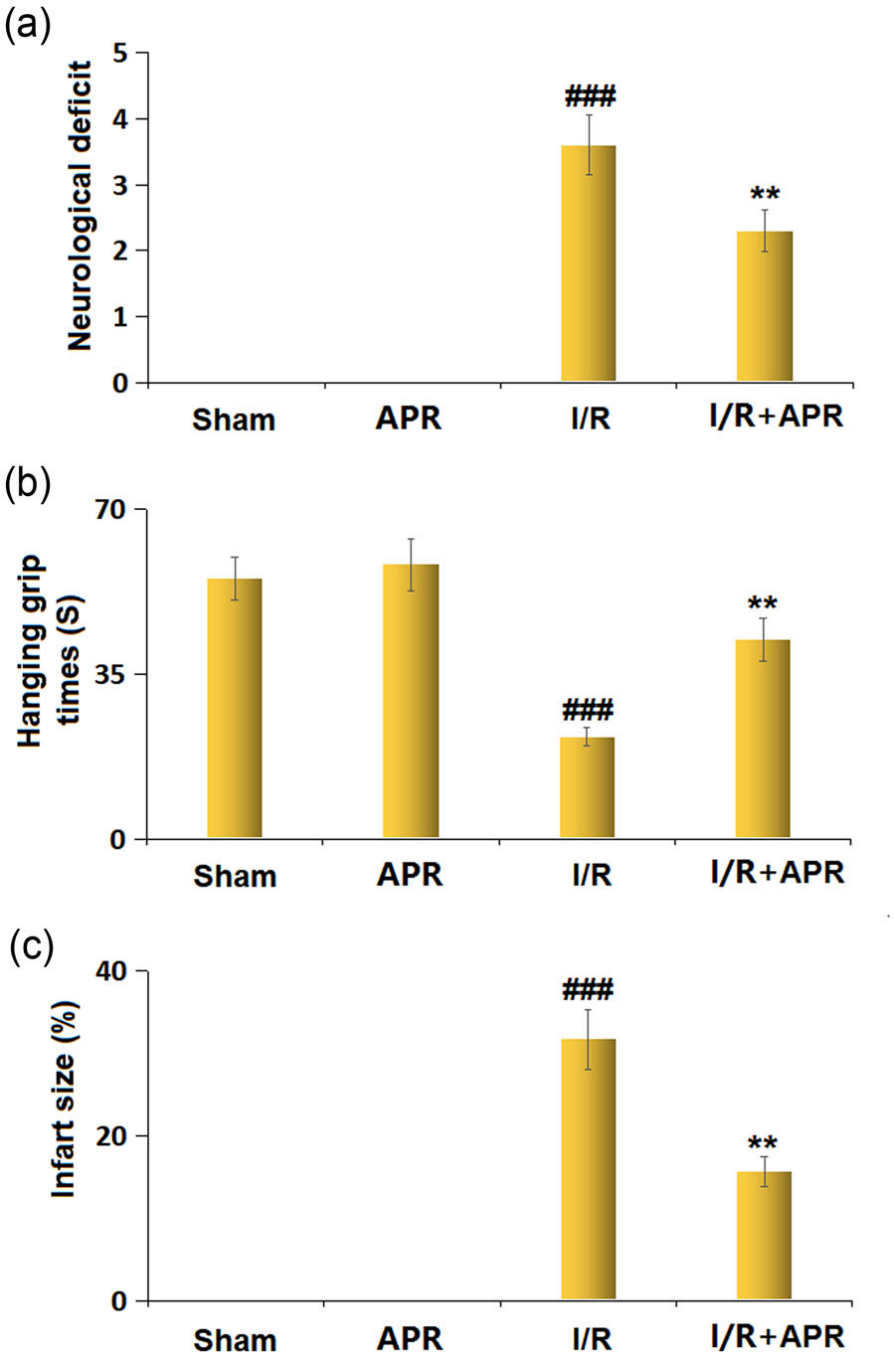
Effects of Apremilast on stroke outcomes. (a) Neurological deficit; (b) hanging grip times; (c) infart size (###*p* < .001 vs. vehicle group; **p < .01 vs. I/R group)

The neurological deficit scores in different groups are shown in Figure 1a. The score in the MCAO group increased when compared to that in the sham group (0). The APR treatment group had a lower neurological deficit score (2.3 ± 0.31) than the MCAO group (3.6 ± 0.45). Compared to the sham group (58.2 ± 5.5 s), mice in the MCAO group exhibited significantly reduced hanging grip time (21.7 ± 1.9 s), which could be attenuated by APR treatment (42.3 ± 4.6 s) (Figure 1b). A significant infarct size was observed in mice from the MCAO group (31.7% ± 3.65%), while it was significantly lessened after APR treatment (15.7 ± 1.8%) (Figure 1c).

### 3.2 Effects of APR on neuroinflammation in the MCAO mice model

**FIGURE 2:**
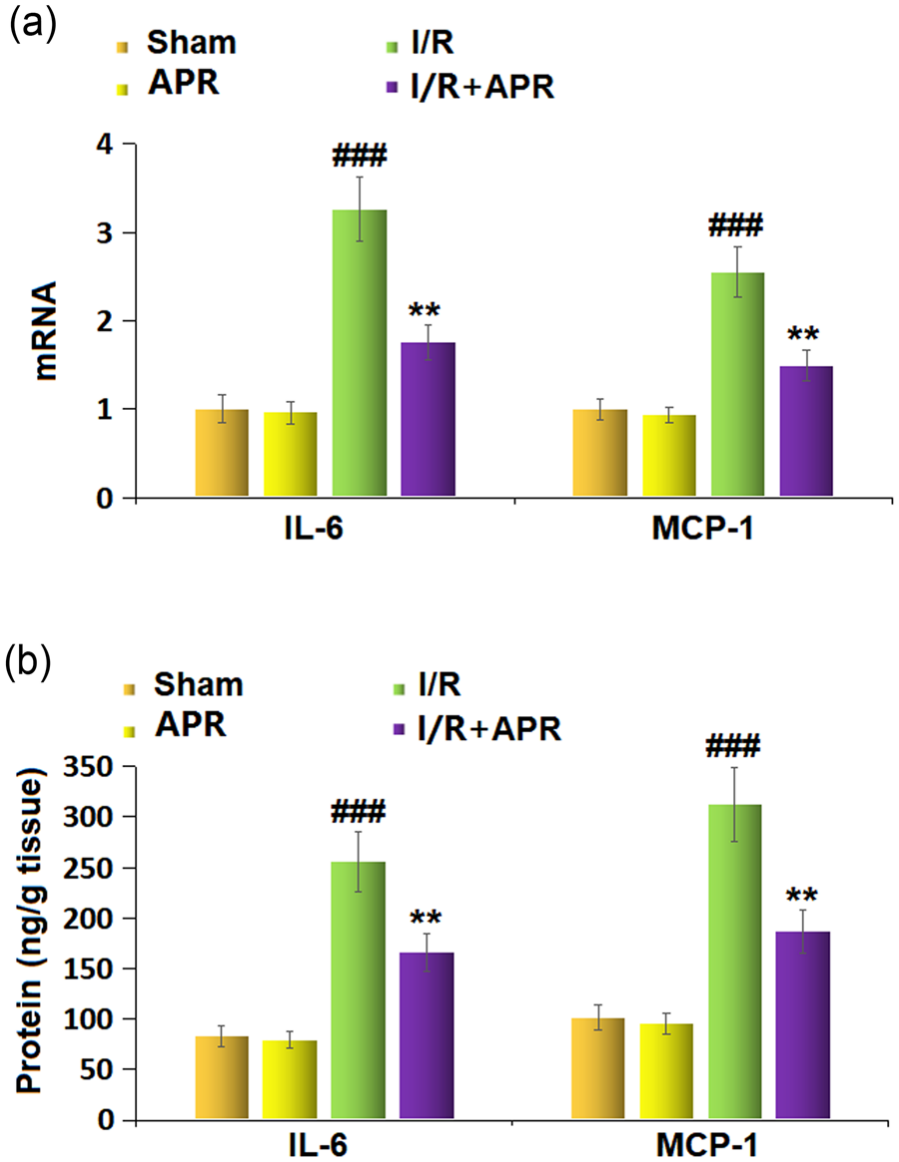
Effects of Apremilast on the expression of proinflammatory cytokines. (a) mRNA of interleukin 6 (IL‐6) and monocyte chemoattractant protein‐1 (MCP‐1); (b) protein levels of IL‐6 and MCP‐1 (###*p* < .001 vs. vehicle group; **p < .01 vs. I/R group) in the cortices of mice as measured by enzyme‐linked immunosorbent assay (ELISA)

Significantly increased mRNA levels of IL‐6 (3.26‐fold) and MCP‐1 (2.55‐fold) were found in the MCAO group compared to the sham group (Figure 2a). However, APR treatment caused significant decreases in both the mRNA levels of IL‐6 and MCP‐1. To support these results, ELISA was carried out to determine the protein levels of IL‐6 and MCP‐1 in the cortex. In the MCAO group, the protein levels of IL‐6 and MCP‐1 were increased to 255.7 ± 29.3 and 312.3 ± 36.8 ng/g tissue, respectively, compared to the sham group (79.6 ± 8.6 and 95.6 ± 10.3 ng/g tissue). However, in the APR treatment group, the elevated protein levels of IL‐6 and MCP‐1 were reduced to 165.7 ± 18.5 and 187.2 ± 21.3 ng/g tissue, respectively.

### 3.3 Effects of APR on BBB disruption in the MCAO mice model

**FIGURE 3:**
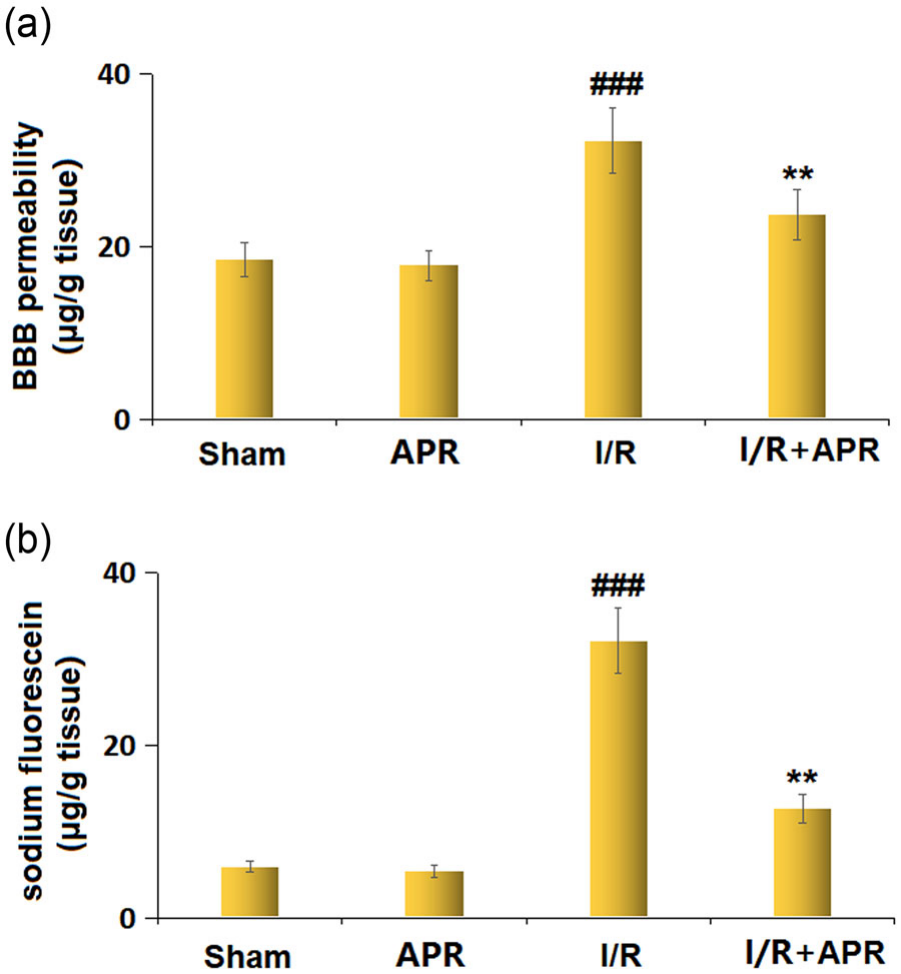
Effects of Apremilast on blood–brain barrier (BBB) disruption in a stroke model. (a) BBB permeability was measured using Evans blue; (b) diffusion of sodium fluorescein in the brains of experimental mice (###*p* < .001 vs. vehicle group; **p < .01 vs. I/R group)

BBB permeability was evaluated using Evans blue and sodium fluorescein leakage in the brains of experimental mice. Figure 3a shows that Evans blue leakage in the brain tissues was markedly higher in the MCAO group (32.3 ± 3.85 μg/g tissue) than in the sham group (17.8 ± 1.68 μg/g tissue); however, this leakage was attenuated by APR treatment (23.7 ± 2.85 μg/g tissue). In addition, the increase in sodium fluorescein leakage in MCAO mice (32.1 ± 3.81 μg/g tissue vs. 5.4 ± 0.72 μg/g tissue in the sham group) was also prevented by APR treatment (12.6 ± 1.65 μg/g tissue) (Figure 3b).

### 3.4 Effects of APR on the expression of Claudin‐5 in the cortices of the MCAO mice model

**FIGURE 4:**
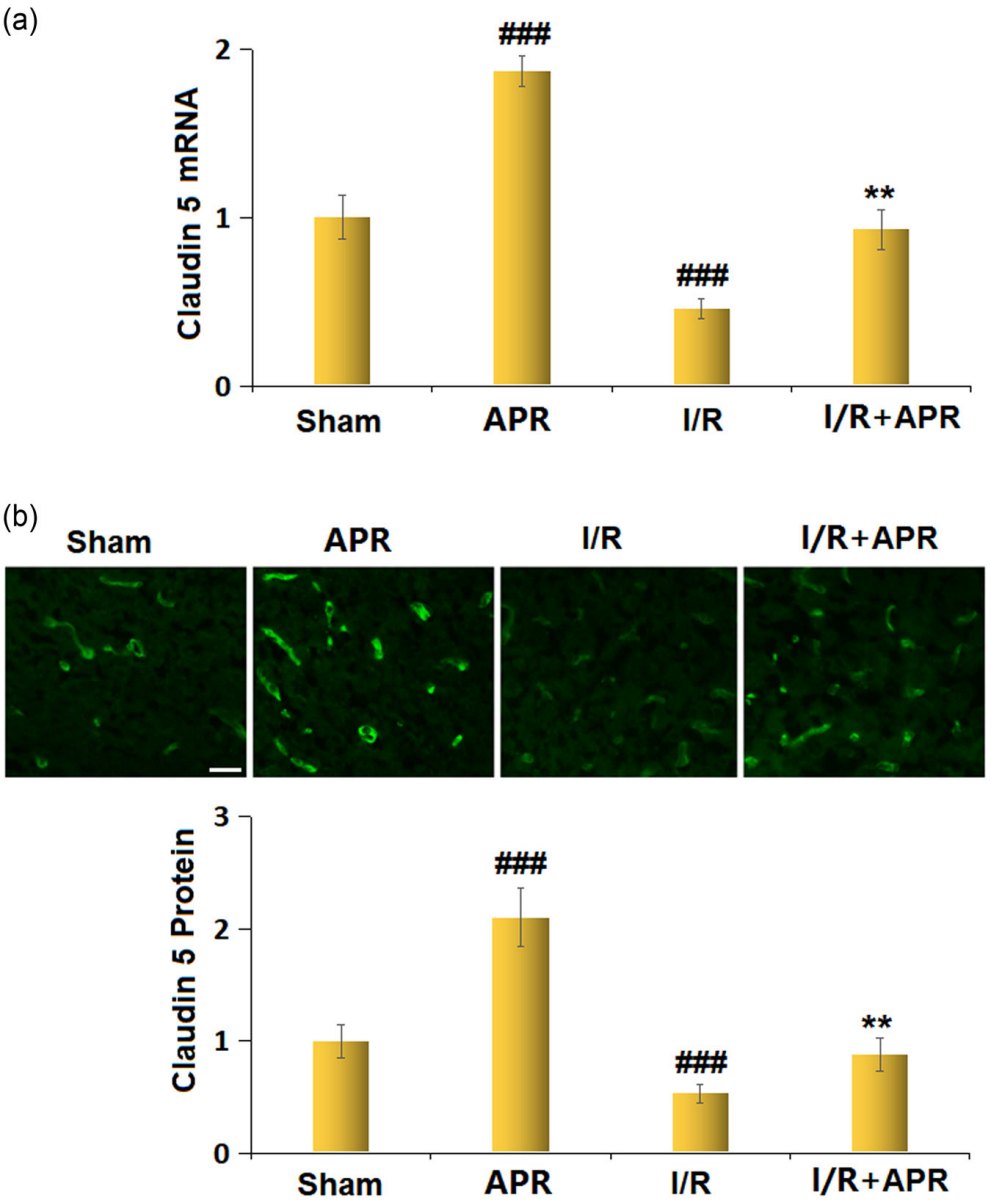
Effects of Apremilast on the expression of Claudin‐5 in the cortex of a stroke mouse model. (a) mRNA of Claudin‐5; (b) protein levels of Claudin‐5 as measured by immunostaining. Scale bar, 50 μm (^###^ *p* < .001 vs. vehicle group; **p < .01 vs. I/R group)

The mRNA levels of Claudin‐5 in the cortex tissues from experimental mice were markedly increased in response to APR treatment alone. Compared to the sham group, mice from the MCAO group presented with decreased mRNA levels of Claudin‐5, which were elevated after APR treatment (Figure 4a). Consistently, the immunostaining assay also showed that APR treatment alone significantly increased the protein level of Claudin‐5. Decreased protein levels of Claudin‐5 were observed in MCAO mice, while they were upregulated by APR administration (Figure 4b).

### 3.5 APR ameliorated the OGD/R‐induced increase in endothelial permeability

**FIGURE 5:**
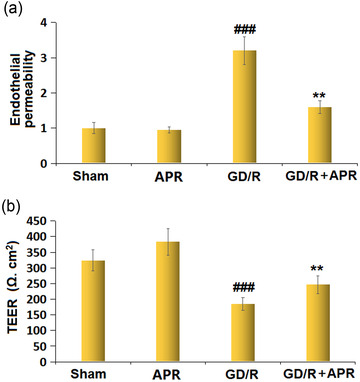
Apremilast ameliorated the oxygen/glucose deprivation/reoxygenation (OGD/R)‐induced increase in endothelial permeability in bEnd.3 brain endothelial cells. (a) Endothelial permeability; (b) transendothelial electrical resistance (TEER) (^###^ *p* < .001 vs. vehicle group; **p < .01 vs. OGD/R group)

We found that sodium fluorescein leakage was markedly increased in OGD/R‐treated bEnd.3 cells. However, it was decreased by intervention with APR (Figure 5a). After OGD/R administration, we observed that the TEER value was significantly decreased in bEnd.3 cells, which could be upregulated by pretreatment with APR (Figure 5b).

### 3.6 APR decreased the expression of Claudin‐5 against OGD/R stimulation in bEnd.3 cells

**FIGURE 6:**
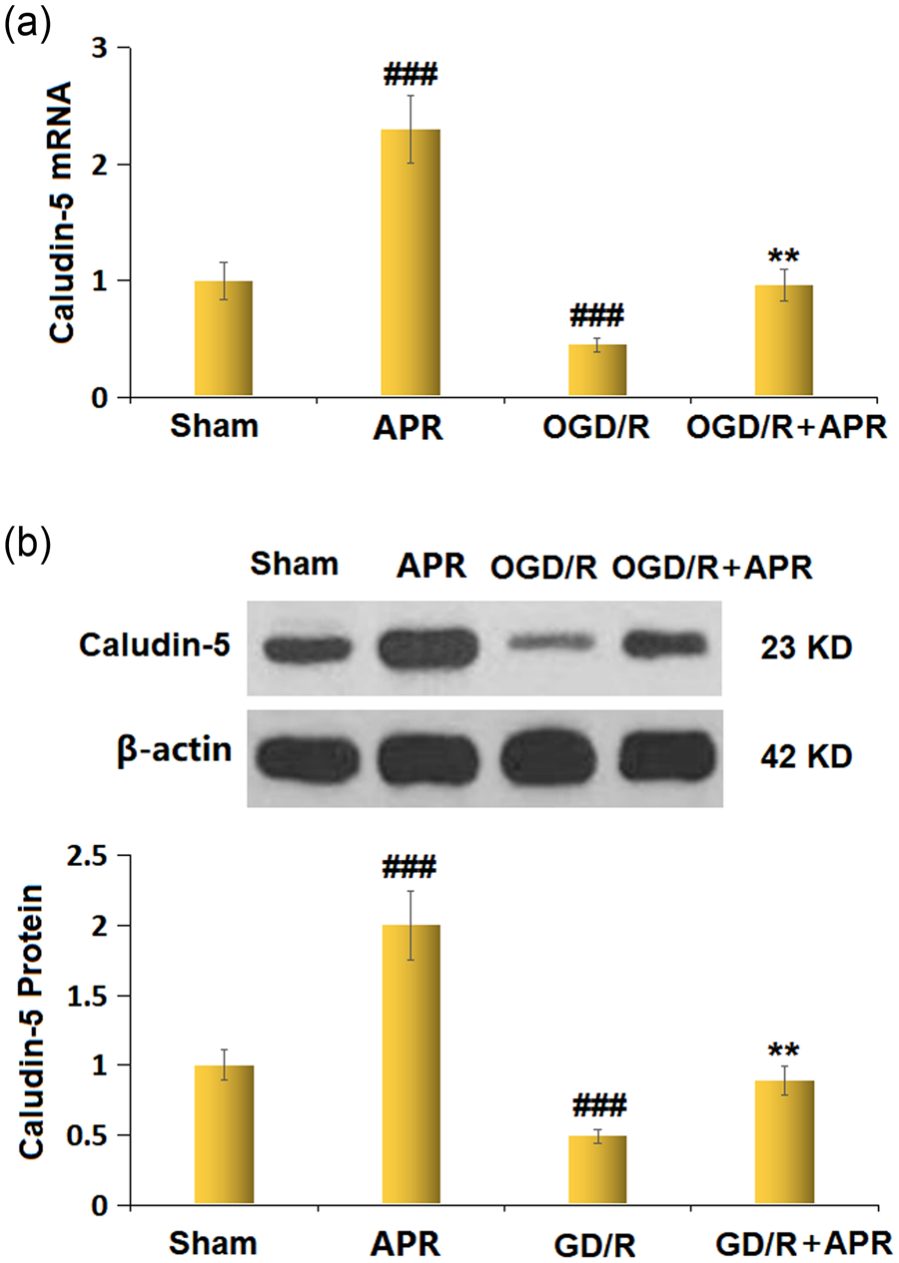
Apremilast restored the expression of Claudin‐5 against oxygen/glucose deprivation/reoxygenation (OGD/R) in bEnd.3 brain endothelial cells. (a) mRNA of Claudin‐5; (b) protein expression of Claudin‐5 (^###^ *p* < .001 vs. vehicle group; **p < 0.01 vs. OGD/R group) as measured by western blot analysis

As shown in Figure 6a, after APR treatment alone, the mRNA level of Claudin‐5 was markedly increased 2.3‐fold. However, the mRNA level of Claudin‐5 in OGD/R‐treated bEnd.3 cells was markedly reduced compared with that in the sham group, and APR pretreatment attenuated this decrease. Consistent with the trend of Claudin‐5 mRNA levels, the protein levels of Claudin‐5 were increased in response to APR treatment alone but decreased in response to OGD/R administration. The decreased protein levels of Claudin‐5 in the OGD/R group were mitigated by APR pretreatment (Figure 6b).

### 3.7 APR reduced the expression of ROCK2 against OGD/R stimulation in bEnd.3 cells

**FIGURE 7:**
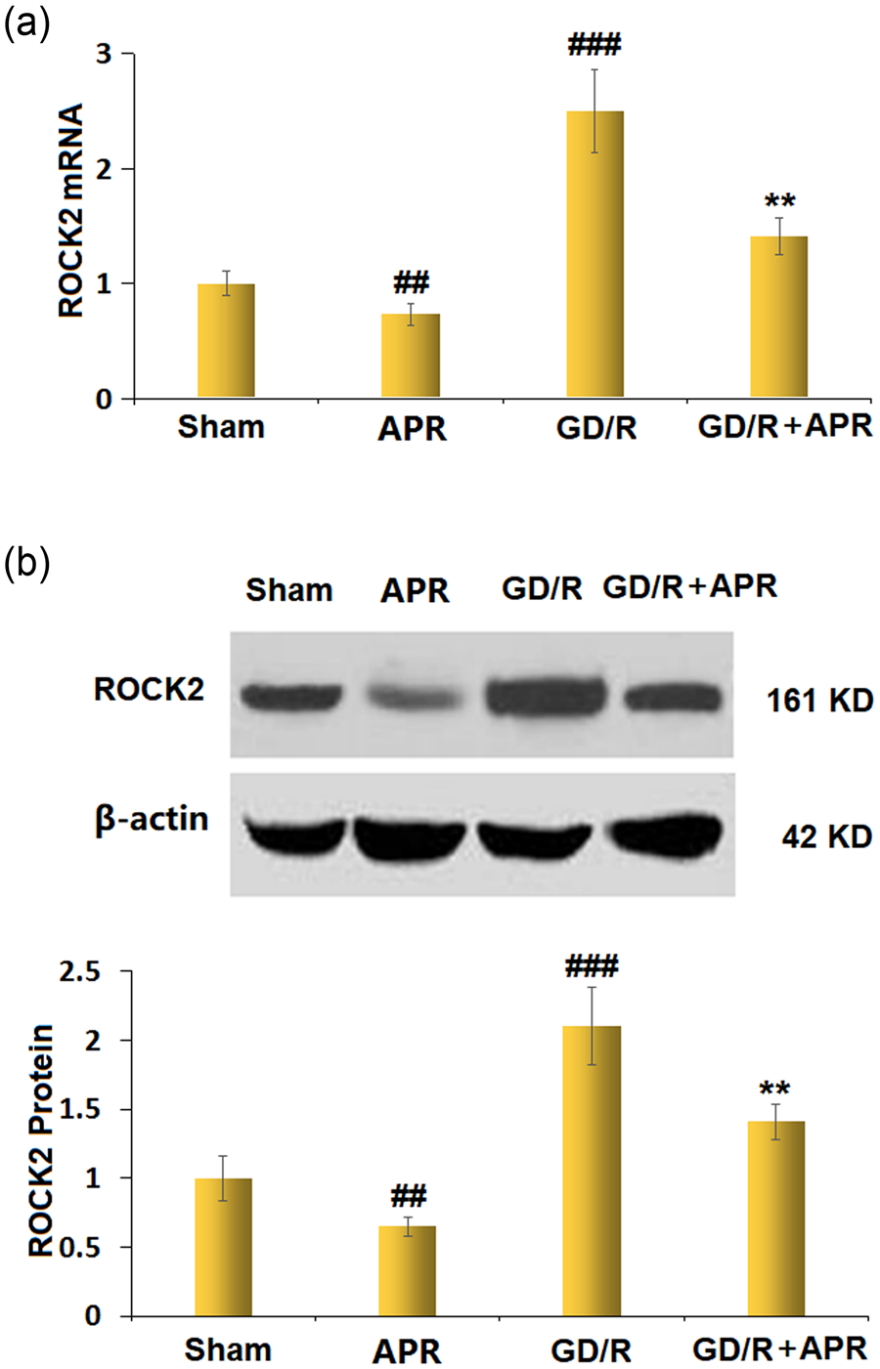
Apremilast reduced the expression of regulating Rho‐associated protein kinase 2 (ROCK2) against oxygen/glucose deprivation/reoxygenation (OGD/R) in bEnd.3 brain endothelial cells. (a) mRNA levels of ROCK2; (b) protein levels of ROCK2 (^##, ###^ *p* < .01, 0.001 vs. vehicle group; **p < .01 vs. OGD/R group)

The mRNA level of ROCK2 in the APR group was distinctly lower than that in the sham group. The OGD/R‐induced increase in ROCK2 mRNA levels was prevented by APR pretreatment (Figure 7a). After incubation with APR, the protein level of ROCK2 declined. Meanwhile, the increased protein level of ROCK2 in OGD/R‐induced bEnd.3 cells was reversed by pretreatment with APR (Figure 7b).

### 3.8 Overexpression of ROCK2 abolished the protective effects of APR on reducing endothelial permeability against OGD/R

**FIGURE 8:**
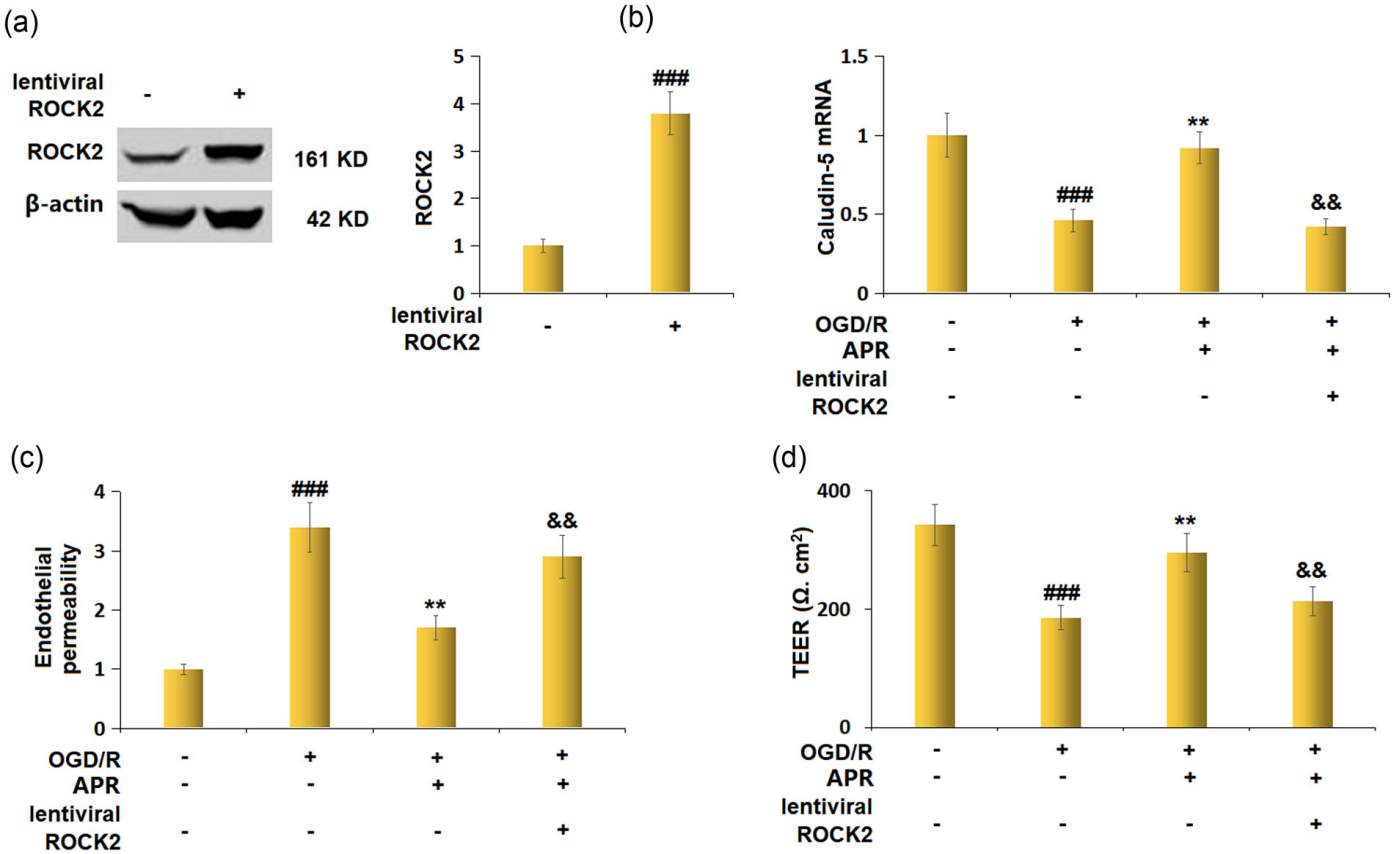
Overexpression of regulating Rho‐associated protein kinase 2 (ROCK2) abolished the protective effects of apremilast in reducing endothelial permeability against oxygen/glucose deprivation/reoxygenation (OGD/R). Cells were infected with lentiviral ROCK2, followed by stimulation with OGD/R in the presence of Apremilast (5 μM). (a) Western blot analysis revealed successful overexpression of ROCK2. (b) mRNA of Claudin‐5; (c) endothelial permeability; (d) transendothelial electrical resistance (TEER) (^###^ *p* < .001 vs. vehicle group; **p < .01 vs. OGD/R group; ^&&^ *p* < 0.01 vs. OGD/R+APR group)

After infection with LV‐NC or LV‐ROCK2, western blot analysis revealed that ROCK2 was successfully overexpressed in LV‐ROCK2‐infected bEnd.3 cells with a 3.8‐fold change (Figure 8a). The APR pretreatment‐induced increase in the mRNA level of Claudin‐5 was prevented by ROCK2 overexpression (Figure 8b). ROCK2 overexpression also disturbed the APR‐induced decrease in endothelial permeability and APR‐induced increase in TEER value (Figure 8c,d).

## 4 DISCUSSION

Recently, some PDE4 inhibitors have been shown to possess protective effects against ischemic stroke. For instance, the PDE4 inhibitor rolipram reduces ischemic stroke severity by reducing BBB disruption, inflammation, apoptosis, and thrombosis in mice (Kraft et al., 2013). Roflumilast (Roflu) is a US Food and Drug Administration‐approved PDE4 inhibitor with therapeutic effects for chronic obstructive pulmonary disease. It attenuates ischemic stroke‐induced neuronal injury by restricting oxidative stress through the glycogen synthase kinase‐3β/nuclear factor‐erythroid 2‐related factor 2 (GSK3β/Nrf‐2) and inositol‐requiring enzyme 1α/TNF‐receptor−associated factor 2/C‐Jun N‐terminal kinase (IRE1α/TRAF2/JNK) pathways (Xu et al., 2021). Chen et al. reported that FCPR16, a synthesized PDE4 inhibitor, possesses protective effects against cerebral ischemia–reperfusion injury in rats through the cyclic adenosine monophosphate/cAMP response element‐binding protein pathway (Chen et al., 2018). Another new synthetic PDE4 inhibitor, FCPR03, was found to alleviate cerebral ischemia/reperfusion injury in neuronal cells and improve brain injury, behavioral performance, and neurological scores in MCAO rats (Xu et al., 2019). Here, we explored the therapeutic potential of APR in protecting against ischemic stroke. The results show that APR attenuated neurological injury in mouse stroke models with decreased neurological deficit scores and infarct size, as well as increased hanging grip time.

Tight junctions among endothelial cells are crucial for restricting the paracellular diffusion of the BBB. Tight junction proteins, including occludins, claudins, and junctional adhesion molecules, join endothelial cells together and directly determine BBB function (Ballabh et al., 2004). In ischemic stroke, tight junction proteins are rapidly degraded, leading to increased BBB permeability. We found that the increased BBB permeability in MCAO mice was attenuated by APR treatment, as evaluated by Evans blue and sodium fluorescein leakage assays. In vitro assays also proved that APR ameliorated the OGD/R‐induced increase in endothelial permeability in bEnd.3 brain endothelial cells. Claudin‐5, an essential tight junction protein, is considered a sensitive indicator of BBB structural integrity (Matter & Balda, 2003). We found that APR treatment alone resulted in a marked increase in the expression levels of Claudin‐5 in cortices from experimental mice and bEnd.3 brain endothelial cells. Intervention with APR in MCAO‐treated mice, as well as OGD/R‐induced bEnd.3 cells, also elevated the expression of Claudin‐5. Collectively, both in vivo and in vitro assays demonstrated that APR treatment attenuated BBB disruption.

In ischemic stroke patients, the immune response is a major factor in pathobiology severity and outcomes. Ischemic stroke is commonly accompanied by a burst of inflammatory reactions due to marked increases in the expression levels of adhesion molecules, cytokines, and other inflammatory mediators, such as prostanoids and nitric oxide (Iadecola & Alexander, 2001). Furthermore, enhanced BBB permeability allows immune cells, such as macrophages and neutrophils, to migrate into the brain parenchyma, leading to neuroinflammation and exacerbation of subsequent edema and brain injury (Anrather & Iadecola, 2016). Our results highlight a critical role of APR in regulating inflammation in MCAO mice, showing that the increased expression levels of the proinflammatory cytokines, IL‐6, and MCP‐1 significantly decreased in the APR treatment group.

**FIGURE 9:**
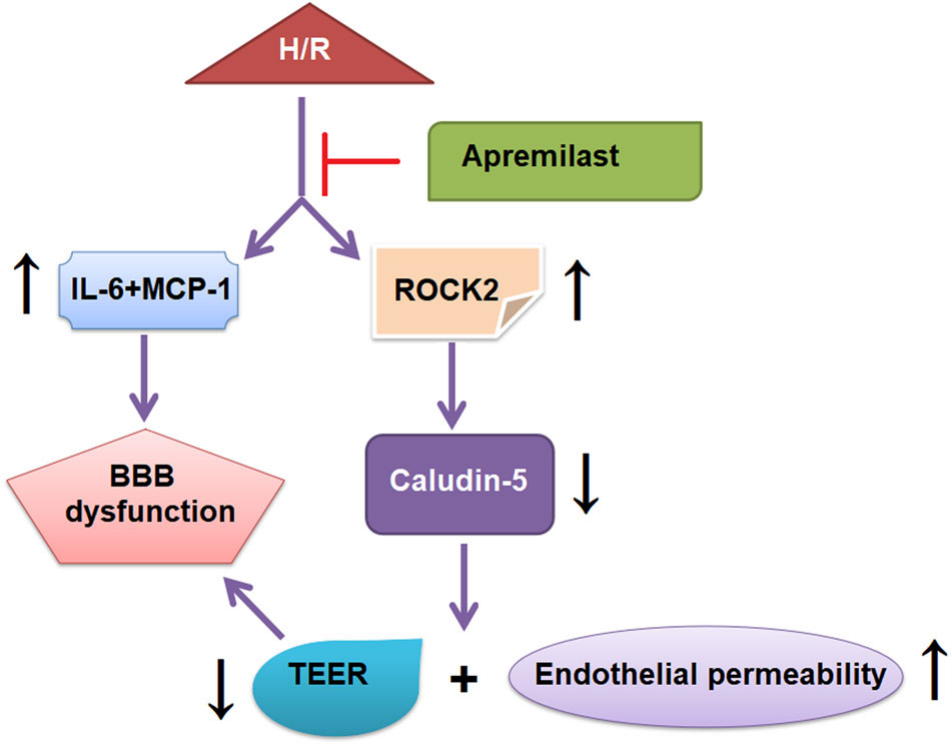
A graphic representation of the underlying molecular mechanism

ROCK2 is a serine/threonine kinase particularly present in brain tissues (Mueller et al., 2005). Previous studies have suggested that ROCK2 performs functional roles in multiple cellular processes, including cell motility, cell adhesion, cell apoptosis, vascular inflammation, and reconstruction (Noma et al., 2006). In addition, ROCK2 expression is subject to changes under pathophysiological conditions (Magro et al., 2020; Qin et al., 2022; Zhou et al., 2020). It has been reported that ROCK2 is connected to diverse cardiovascular diseases, including atherosclerosis, hypertension, cardiac hypertrophy, and ischemic stroke (Lee et al., 2014; Okamoto et al., 2013; Takeda et al., 2019; Yamamura et al., 2021). Regarding its physiological role in the cardiovascular system, it is speculated that inhibition of ROCK2 may provide cardiovascular benefits. Here, we found that APR treatment alone caused significantly reduced expression of ROCK2 in bEnd.3 cells. The OGD/R‐induced increase in ROCK2 expression was prevented by APR pretreatment. Moreover, overexpression of ROCK2 in bEnd.3 cells abolished the protective effects of APR on endothelial permeability against OGD/R induction, implying that APR exerted its roles via regulating ROCK2. A graphic representation of the underlying molecular mechanism is shown in Figure 9.

In summary, the novel PDE4 inhibitor APR showed significant efficacy on ischemic stroke outcomes by alleviating enhanced BBB permeability and neuroinflammation by inhibiting ROCK2. These findings suggest a novel therapeutic window for ischemic stroke.

## CONFLICT OF INTEREST

The authors declare no conflict of interest.

## AUTHOR CONTRIBUTIONS

Mingyuan Wang and Zhihua Cheng designed the study. Mingyuan Wang and Xiangyuan Meng performed the study. Zhihua Cheng prepared the manuscript. All the authors have read and approved the submission.

### PEER REVIEW

The peer review history for this article is available at https://publons.com/publon/10.1002/brb3.2677

## Data Availability

The data that support the findings of this study are available from the corresponding author upon reasonable request.
